# Integrative Systems Biology Investigation of Fabry Disease

**DOI:** 10.3390/diseases4040035

**Published:** 2016-11-15

**Authors:** Marco Fernandes, Holger Husi

**Affiliations:** Institute of Cardiovascular and Medical Sciences, BHF Glasgow Cardiovascular Research Centre, University of Glasgow, 126 University Place, Glasgow, G12 8TA, UK; m.fernandes.1@research.gla.ac.uk

**Keywords:** Anderson-Fabry Disease, omics, data integration, disease modelling

## Abstract

Fabry disease (FD) is a rare X-linked recessive genetic disorder caused by a deficient activity of the lysosomal enzyme alpha-galactosidase A (GLA) and is characterised by intra-lysosomal accumulation of globotriaosylceramide (Gb3). We performed a meta-analysis of peer-reviewed publications including high-throughput omics technologies including naïve patients and those undergoing enzyme replacement therapy (ERT). This study describes FD on a systems level using a systems biology approach, in which molecular data sourced from multi-omics studies is extracted from the literature and integrated as a whole in order to reveal the biochemical processes and molecular pathways potentially affected by the dysregulation of differentially expressed molecules. In this way new insights are provided that describe the pathophysiology of this rare disease. Using gene ontology and pathway term clustering, FD displays the involvement of major biological processes such as the acute inflammatory response, regulation of wound healing, extracellular matrix (ECM) remodelling, regulation of peptidase activity, and cellular response to reactive oxygen species (ROS). Differential expression of acute-phase response proteins in the groups of naïve (up-regulation of ORM1, ORM2, ITIH4, SERPINA3 and FGA) and ERT (down-regulation of FGA, ORM1 and ORM2) patients could be potential hallmarks for distinction of these two patient groups.

## 1. Introduction

Fabry disease (FD) is a rare X-linked recessive genetic disorder caused by a deficient activity of the lysosomal enzyme alpha-galactosidase A (GLA). It is characterised by intra-lysosomal accumulation of globotriaosylceramide (Gb3) and other related glycolipids sub-types (e.g., glycosphingolipids) in many cell types, including blood vessels throughout the body. This buildup initiates a cascade of events, starting with the disruption of basic metabolic processes at the cellular level and progressing to inflammatory events and cell death, thereby affecting the system as a whole with an increase of renal, cardiac, cerebrovascular, and skin complications [1,2].

FD is associated with vascular injury and a high recurrence rate of thrombotic events, caused by decreased levels of thrombomodulin (TM) and increased levels of plasminogen activator inhibitor (PAI), both playing a role in the regulation of the endothelium and leukocyte interactions [3].

The impairment of the endothelium-dependent vascular dilation triggered by abnormal levels of nitric oxide (NO) and aberrant activity of the endothelial nitric oxide synthase (eNOS) are known factors associated with an increased incidence of stroke amongst Fabry patients [4].

In comparison to other lysosomal storage disorders, FD is one of the more common types and occurs in approximately 1 in 40,000 male births [2]. When racial origins are taken into account, the majority of the patients are Caucasians; however, it is also found in African Americans and in subjects of Asian ancestry [5].

As occurs in other X-linked inherited disorders, males with deleterious mutations have slight to no residual alpha-galactosidase A activity. Consequently, these patients experience the full range of disease symptoms. The disease presentation in female carriers is more variable and depends on the normal-to-mutant ratio of GLA within the different tissues of the body [5,6,7].

Historically, the average life span of a male patient with FD was around 41 years, if left untreated, which can be substantially improved by kidney transplants, dialysis, and, more lately, enzyme replacement therapy (ERT). Thus, at the time, renal failure, heart failure, and stroke were the most common causes of death [6,7]. FD management and therapy employs the regular use of agalsidase-alpha (Replagal) and agalsidase-beta (Fabrazyme). They are reported as improvers of renal and cardiac function, and cerebrovascular flow [1,8].

The introduction of enzyme replacement therapy for FD posed a major advance in the treatment of lysosomal storage diseases [9], but monitoring the effects of enzyme replacement therapy in the clinical practice is challenging as there are no robust surrogate markers of short-term response [10].

Mouse GLA shares a high homology with human GLA; therefore, the GLA-knockout mouse model of FD has been widely used in studies performing microarray gene expression analysis [11,12]. The FD mouse model presents a normal adult lifespan with normal blood and urine analysis, but has a number of limitations compared to the human disease including fewer symptoms with no evident histological lesions in stained sections of the kidneys, liver, heart, spleen, lungs and brain compared to humans, even though this animal model mimics the main neurological features of FD, including reduced locomotor activity, impairment of balance and coordination and diminished sensitivity to painful stimuli. On the other hand, the animal model shows progressive and demarcated accumulation of Gb3 in the kidneys and liver which makes the mouse model suitable for the development of enzyme replacement therapies via the assessment of the clearance of accumulated Gb3 in the affected organs [13].

During the past decade, major advances in the field of omics technologies have led to an exponential growth in available experimental data. Most of the omics platforms provide high-throughput, detailed exploration of the genome, transcriptome, proteome and metabolome through a variety of techniques comprising microarrays for mRNA and miRNA, next-generation sequencing and mass spectrometry [14].

The intrinsic nature of omics data being noisy and usually on a very large scale causes additional problems during analysis and concomitantly gives rise to other layers of complexity that are added due to the biological context, which is often highly diverse [15].

Finding value in complex biological data streams, such as in high-throughput omics platforms, is the upcoming era for the use of combinatorial integrative approaches using bioinformatics resources and systems biology methodologies. Thus, the latter approach has the potential to promote an unbiased and knowledge-driven platform for hypothesis generation in the context of disease modelling. As a consequence, traditional methods using hypothesis-driven investigations in the study of complex human disorders are converging to a hypothesis-free systems approach to exploit the potential to yield an unbiased and novel testable hypothesis as an end-result.

One of the main gaps of the current “panorama” is the disparity between the fast generation of large-scale data and their in-depth analysis. Hence, a high volume of omics data has been generated with the goal to discover novel disease biomarkers. Although it found its way into publicly available databases, no effort has been made to combine and consolidate these resources in order to offer an integrative view of all studies.

Omics studies and publications concerning Fabry disease are scarce and even more so for the availability of the underlying raw data. Thus, using the end-results of publications, i.e. tables and lists containing detected molecules, fold-changes in disease, statistical scores and other relevant molecular information acquired from omics discovery platforms as data source for a further meta-analysis, appears to be an alternative to fulfil the absence of native FD datasets.

Here, we describe FD at a systems level using a systems biology approach, in which molecular data sourced from multi-omics studies was extracted from the literature and integrated as a whole, in order to reveal the biochemical processes and molecular pathways potentially affected by the dysregulation of differentially expressed molecules and in this way provide new insights that describe the pathophysiology of this rare disease.

## 2. Materials and Methods

### 2.1. Data Mining

**Figure 1:**
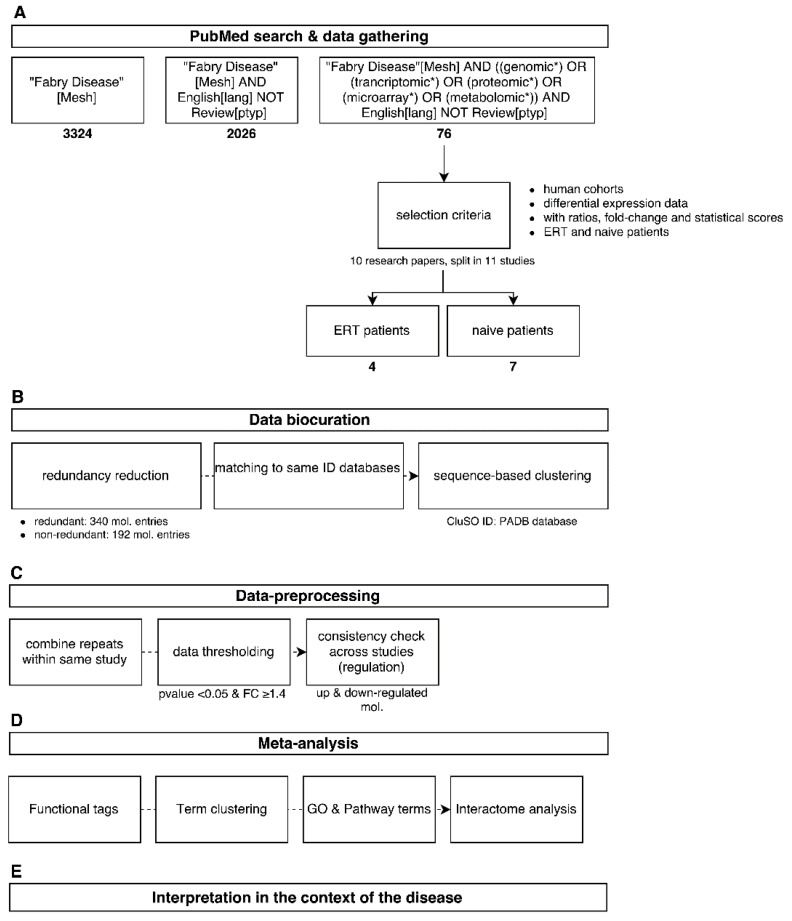
Overview of the analysis workflow used in this study from data mining (**A**) to global interpretation of the results (**E**). Data was acquired from the literature (**A**); followed by data curation and harmonization (**B**); Analysis was performed by dataset merging and application of thresholds (**C**); computational methods (**D**) and combination of all results (**E**).

Several search strings were used in the PubMed literature database in order to cover the topic of Fabry disease and omics as much as possible using, for example, "Fabry[Mesh] AND (miRNA OR genomic* OR proteomic* OR metabolomic*) NOT review” (Figure 1A). After the initial search effort, 76 research papers were retrieved and a preliminary selection criteria was applied taking in account only papers dealing with human cohorts that made use of a omics biomarker discovery platform, such as mass spectrometry technologies and microarrays. Publications that focused solely on characterising one molecule a time were not included. Molecular data concerning differential expression profiling were gathered from the main body of the literature itself or by using lists and tables provided in the supplementary material section. Ratios and fold-changes associated with individual molecules and as well their statistical scores were retained. This resulted in four research publications characterising molecular differential expression in patients undergoing enzymatic replacement therapy (ERT) and seven scientific investigations characterising molecular differential expression in FD-naïve patients (Figure 1A).

### 2.2. Data Biocuration

The step of data curation plays a central role in database development as well as in a systems analysis, for which the quality of the collected data is verified by comparing it with similar studies and by assessing the relevance of the parameters used in the detection of a molecule or molecule population, such as statistical thresholds, normalisation method, applied cut-offs, and validation steps. Reference databases used for matching an initial cross-platform dataset of 340 redundant molecule entities were retrieved and mapped to a non-redundant molecular cluster database (CluSO, the clustered sequences and orthologs database resource from the Pan-omics Analysis Database (PADB) initiative). This resulted in 192 non-redundant molecule entities (Figure 1B). Additionally, these identifiers enable a unified and harmonised data-linkage that allows dealing with the high heterogeneity of the data sourced from multi-omics studies.

Only statistically significant and differently expressed molecules were retained for further processing (*p*-value < 0.05, and fold-changes (FC), in which the selected threshold is dependent on the detection method used by the authors, i.e. transcriptomics FC ≥ 2; proteomics and metabolomics FC ≥ 1.3).

### 2.3. Data Pre-Processing

In order to get a focused overview of the associated biological processes and pathways associated with Fabry disease, molecular information detected in at least two tissues/fluid sources using a multitude of -omics platforms captured in 11 human studies were taken forward (Table 1, for dataspace description). This was followed by combining redundant entries within the same study by taking the mean of the expression ratios of repeated molecules. Then, a global threshold of 1.4 as fold-change (a 40% increase or decrease of the molecular expression regarding case and control ratios) and a *p*-value < 0.05 was applied. Subsequently, a consistency check based on the directionality (down-regulated if <1.4 and up-regulated if >1.4) of the molecular expression profiles across different studies was applied in order to remove entries with contradictory regulation and merge consistent entries by averaging their ratios (Figure 1C).

### 2.4. Meta-Analysis

We performed functionality tag clustering, statistical gene ontology (GO) and pathway term clustering, molecular clustering based on protein-protein interactions (PPIs), mapping of the molecular features into existing pathway maps and the description of putative regulatory interactions (Figure 1D). Functionalities were obtained from the CluSO database, whereby every single molecular cluster is assigned a specific deprecated global functionality tag based on a hierarchical system.

GO and pathway term clustering was done using ClueGO v2.2.5 [16] and Cytoscape (version 3) [17]. GO analysis was done using the GO categories: biological process, molecular function, cellular component, and pathway descriptors derived from Reactome [18], KEGG (Kyoto Encyclopedia of Genes and Genomes) [19] and WikiPathways [20]. We applied the two-sided hypergeometric test as the statistical procedure with Bonferroni step-down correction, kappa score threshold of 0.4 and *p*-value enrichment cut-off of 0.05.

Molecular clustering involved the use of GeneMania [21] as a Cytoscape application [17]. Pathway mapping was achieved using PathVisio (version 3 [22] and a collection of reference pathway maps. Statistical evaluation included the built-in statistical module using a Z-score cut-off of 1.6449.

We provided the association between gene and disease phenotypes of the most frequently reported molecules in our dataset for both naïve and ERT groups by using DisGeNET version 2.1 [23], an app for Cytoscape, which gathers supportive evidence from several public resources and by text-mining the literature in order to rank gene-disease associations based on our initial queries.

For a view over potential up-stream regulators, we used the CyTargetLinker [24] app for Cytoscape which displays regulatory elements such as miRNA-target and transcription factor (TF)-target as a network based on our initial queries. The CyTargetLinker app uses regulatory information from the Regulatory Interaction Networks (RegINs).

### 2.5. Interpretation in the Context of the Disease

The final step in the analysis described here involved the merging of results and the overall interpretation at a systems level, within which individual results from a multitude of bioinformatics tools are combined in order to yield biological meaning of the analysis performed (Figure 1E).

## 3. Results

### 3.1. Dataspace Description

The datasets extracted from 11 studies sourced from ten research papers and then used in our meta-analysis are described in Table 1. It handles group modifiers such the description of the underlying disease and therapies, in addition to cohort number both as a total and split by case and control scenarios. Moreover, information of the detected molecules in several multi-omics platforms in two fluid sources are also handled.

### 3.2. Functionality Tag Clustering

**Figure 2:**
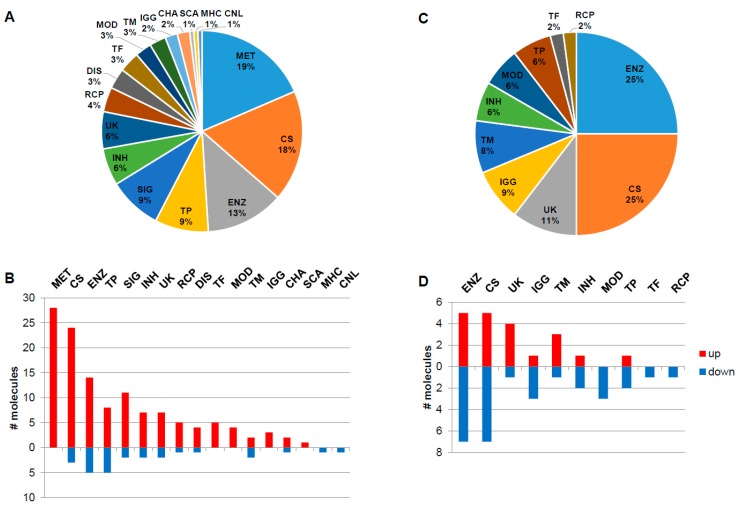
Functionality tag clustering of the total differentially expressed molecules (N = 151, fold-change ≥ 1.4 and *p*-value < 0.05) reported in the literature for the naïve group (**A**). Associated tags for deregulated molecules: down (N = 26) and up-regulated (N = 125) molecules (fold-change ≥ 1.4 and *p*-value < 0.05) reported in the literature for the naïve group (**B**). Functionality tag clustering of the total differentially expressed molecules (N = 48, fold-change ≥ 1.4 and *p*-value < 0.05) reported in the literature for the ERT group (**C**). Associated tags for deregulated molecules: down (N = 28) and up-regulated (N = 20) molecules (fold-change ≥ 1.4 and *p*-value < 0.05) reported in the literature for the ERT group (**D**). MET: metabolite; CS: Cell shape (cytoskeleton, cell adhesion, morphology, cell junction, cellular structures, extracellular matrix); ENZ: enzyme, enzymatic properties; TP: transport, storage, endocytosis, exocytosis, vesicles; SIG: signalling; INH: inhibitor (protease, kinase, other enzymes, pathways); UK: unknown; RCP: receptor; DIS: disease; TF: transcription and translation, gene regulation; MOD: modulator, regulator; TM: transmembrane; IGG: Immunoglobulin; CHA: chaperone, chaperonin; SCA: scaffolder, docking, adaptor; MHC: major histocompatibility complex component/protein cluster (MHC, HLA); CNL: channel.

The naïve group contains globally around five times more overexpressed molecular features with increased expression levels than decreased, while in the ERT group the number of down- and up-regulated features is nearly identically distributed (Figure 2). The main functionality tags accounting for more than 7% and detected in the naïve group were 19%–MET: metabolite; 18%–CS: Cell shape (cytoskeleton, cell adhesion, morphology, cell junction, cellular structures, extracellular matrix); 13%–ENZ: enzyme, enzymatic properties; 9%–TP: transport, storage, endocytosis, exocytosis, vesicles and 9%–SIG: signalling molecules (Figure 2A). In the naïve group, the molecules associated with the functionality tags of MET: metabolite, TF: transcription and translation, gene regulation, MOD: modulator, regulator, IGG: Immunoglobulin, and SCA: scaffolder, docking, adaptor, were found to be exclusively up-regulated, in contrast to the MHC: major histocompatibility complex and CNL: channel tags that down-regulated (Figure 2B). In the ERT group, 25%–ENZ: enzyme, enzymatic properties; 25%–CS: Cell shape (cytoskeleton, cell adhesion, morphology, cell junction, cellular structures, extracellular matrix); 11%–UK: unknown; 9%–IGG: Immunoglobulin; and 8%–TM: transmembrane molecules were detected (Figure 2C). In the ERT group, the molecules associated with the functionalities tags of MOD: modulator, regulator, TF: transcription and translation, gene regulation, and RCP: receptor were found exclusively to be down-regulated (Figure 2D).

This primary approach is helpful in that it provides basic information characterising the functional role of the molecules within the combined datasets. Therefore, such an approach helps to determine the directionality of further downstream analysis steps, and whether particular areas—e.g., regulatory networks (in case of transcriptional factors, miRNAs and their target genes), signalling pathways, metabolic pathways, and so forth—are more likely to be involved in the disease.

### 3.3. Expression Correlation Across Fabry Studies and Most Frequent Reported Molecules

**Figure 3:**
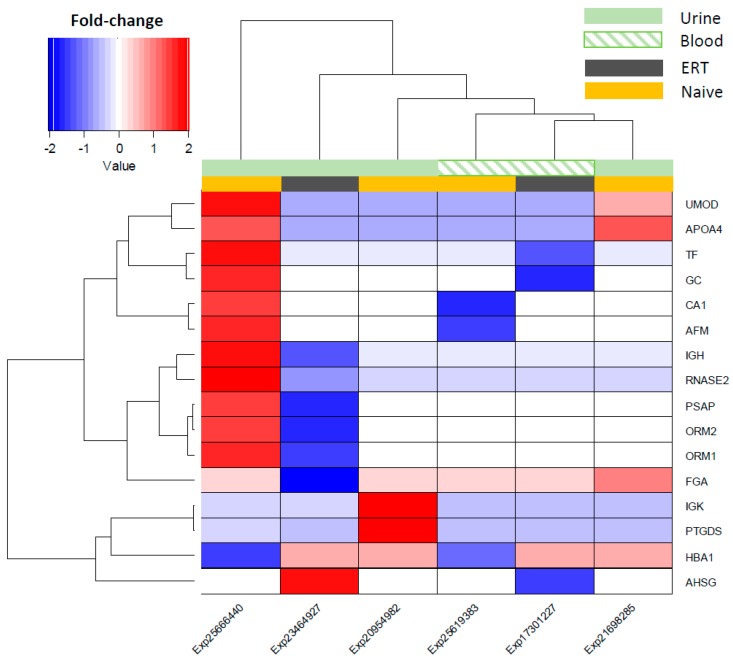
Hierarchical clustering of the differential molecular expression across Fabry studies and by fluid source. Decreased expression shown in a blue and increased expression by the red colour gradient. Group modulation for the urine fluid source is shown as light green in a block, whereas for the blood it is presented as a light green pattern. Group modulation representing the type of treatment is shown as dark-grey blocks for ERT patients and light-orange blocks for the non-treated patients (naïve group).

Hierarchical clustering was performed using fold-change values of the overlapping features (16 in total) across all Fabry studies in naïve and ERT patients, and the source fluids (urine and blood) which they were detected in as group modifiers (Figure 3). Overall, the expression of the overlapping molecules across Fabry studies points to an overexpression in the naïve group and a decrease for the ERT group (Figure 3). It can be observed that the studies Exp25666440 and Exp23464927, from the naïve and ERT groups, respectively, and both detected in urine, show a molecular expression pattern that is quite distinct from the other samples/studies. Based on the similarity of the molecular expression, the detected features can be split into two main clusters: one containing IGK, PTGDS, HBA1, and AHSG, and another containing UMOD, APOA4, TF, GC, CA1, AFM, GH, RNASE2, PSAP, ORM2, ORM1, and FGA (Figure 3). A list with the most frequently reported and consistently regulated molecules in Fabry-naïve and ERT groups can be found in the Supplementary Tables S1 and S2.

### 3.4. Gene Ontology (GO) Term Clustering

**Figure 4:**
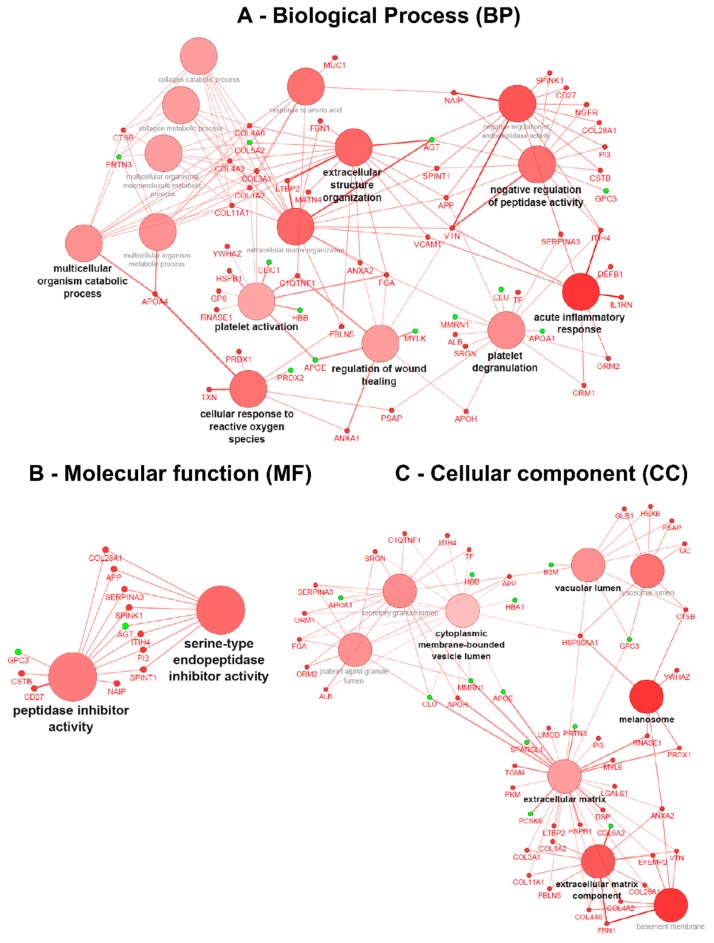
Gene ontology (GO) term clustering (all data) of the naïve group: (**A**) Biological process (BP), (**B**) Molecular function (MF) and (**C**) Cellular component (CC). The increase of node size is associated with an increase of the statistical significance (Bonferroni-corrected *p*-value), the red node colour denotes an increased regulation of the term/group and green a decrease. Network nodes displayed in grey means that they share an equal number of genes/proteins associated with an up- and down-regulation.

**Figure 5:**
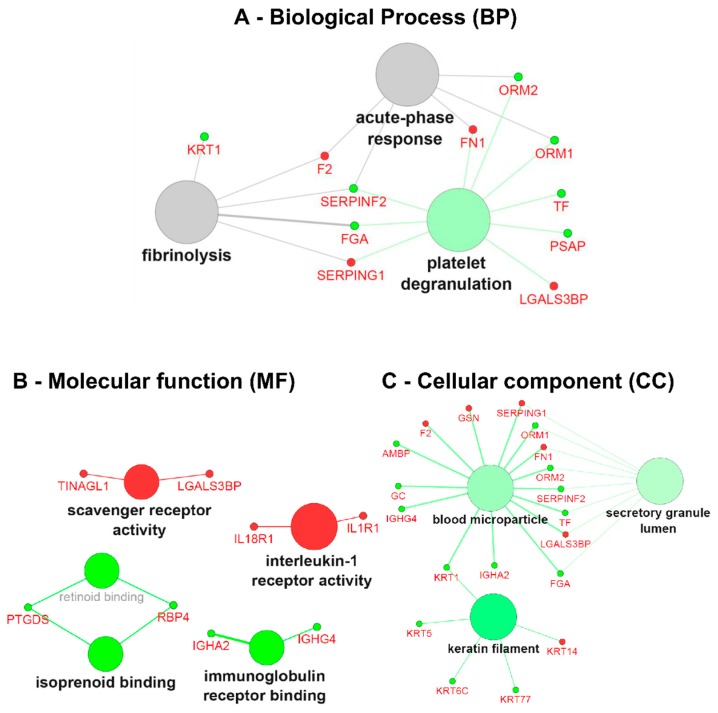
Gene ontology (GO) term clustering (all data) of the ERT group: (**A**) Biological process (BP), (**B**)Molecular function (MF) and (**C**) Cellular component (CC). The increase of node size is associated with an increase of the statistical significance (Bonferroni-corrected *p*-value), the red node colour denotes an increased regulation of the term/group and green a decrease. Network nodes displayed in grey means that they share an equal number of genes/proteins associated with an up- and down-regulation.

After applying data thresholding (*p*-value < 0.05 and Fold-change ≥ 1.4) across the entire dataspace, the Cytoscape plugin ClueGO v.2.2.5 [16] was used to identify the associated biological processes (BP), molecular functions (MF) and cellular components (CC) using gene ontology (GO) for both naïve and ERT groups, taking into account overexpressed and under-expressed molecules (Figure 4 and Figure 5). Pathway term clustering using Reactome, KEGG and WikiPathways can be found in the Supplementary Figures S1–S5, and Tables S3 and S4, respectively.

In the GO term analysis of the naïve group, it was found that all the described biological processes were activated, meaning that the majority of the associated genes with the GO term were overexpressed (Figure 4A). The process of acute inflammatory response was found to be activated with all the genes belonging to cluster #1 (Table S3); DEFB1, IL1RN, ITIH4, ORM1, ORM2, SERPINA3, VCAM1 and VTN increased in expression. In a similar way, the C1QTNF1, COL1A2, COL3A1, FGA, GP6, HSPB1, RNASE1, and YWHAZ genes associated with platelet activation were also overexpressed,thereby indicating that the process itself is most likely activated too. The subsequent process of granule secretion by the platelet (platelet degranulation) involved ALB, APOH, APP, FGA, ITIH4, ORM1, ORM2, PSAP, SERPINA3, SRGN, and TF genes, and was also found triggered by the overexpression of the associated genes. Concomitantly, the wound-healing process was found activated with the genes belonging to cluster #1 (ANXA1, ANXA2, APOH, C1QTNF1, FGA, and VTN) being increased in expression. The process of regulation of peptidase activity was found activated with the genes belonging to cluster #1 (APP, CD27, COL28A1, CSTB, ITIH4, NAIP, NGFR, PI3, SERPINA3, SPINK1, SPINT1, and VTN) being increased in expression. Moreover, by the analysis of the GO molecular function, this regulation is driven by peptidase inhibitors belonging to the serine type (Figure 4B).

The process of cellular response to reactive oxygen species (ROS) was found activated with the genes belonging to cluster #1 (ANXA1, APOA4, FBLN5, PRDX1, PSAP, and TXN) being increased in expression. The process of extracellular matrix organisation (ECM) was found activated with the genes belonging to cluster #1 (ANXA2, APP, COL11A1, COL1A2, COL3A1, COL4A2, COL4A6, FBLN5, FBN1, FGA, LTBP2, MATN4, SPINT1, VCAM1, and VTN) being increased in expression. Likewise, the child GO term of the multicellular organism catabolic process for which the process is driven by collagen synthesis (collagen catabolic process) involves COL11A1, COL1A2, COL3A1, COL4A2, COL4A6, and CTSB genes also found to be activated (Figure 4A).

Regarding the GO term associated with the cellular component for the naïve group, it could be demonstrated that overexpressed genes are located at the extracellular matrix, such as ANXA2, APOH, COL11A1, COL1A2, COL28A1, COL3A1, COL4A2, COL4A6, DSP, EFEMP2, FBLN5, FBN1, HSP90AA1, HSPB1, LGALS1, LTBP2, MYL6, PI3, PKM, PRDX1, RNASE1, TGM4, UMOD, and VTN. Located at the melanosomes with ANXA2, the CTSB, HSP90AA1, PRDX1, RNASE1, and YWHAZ genes increased in expression. Additionally, basement membrane (basolateral plasma membrane)-associated molecules ANXA2, COL28A1, COL4A2, COL4A6, EFEMP2, FBN1, and VTN also increased in expression, as did vacuolar lumen-located molecules APP, CTSB, GC, GLB1, HEXB, HSP90AA1, PSAP (Figure 4C). A more detailed view of the GO terms associated with the genes/protein for the naïve group can be found in Supplementary Table S3.

In the GO term analysis of the ERT group, the process of platelet degranulation was found to be inactivated with the genes belonging to cluster #2 (Table S4); FGA, ORM1, ORM2, PSAP, SERPINF2, TF decreased in expression. Biological processes such as the acute-phase response and fibrinolysis were not found to be significantly modulated in between groups, though significantly modulated for each group, taking into account the cross-correlation of the two initial clusters (cluster #1: up-regulated genes, and cluster #2: down-regulated genes) since both shared a similar number of up- (~40%) and down-regulated (~60%) genes (Figure 5A and Supplementary Table S4).

The GO term analysis of the molecular function (MF) revealed the involvement and activation of scavenger receptor activity (TINAGL1 and LGALS3BP), interleukin-1 receptor activity (IL18R1 and IL1R1), and on the other hand inactivation of isoprenoid binding (PTGDS and RBP4) and immunoglobulin receptor binding (IGHA2 and IGHG4) (Figure 5B). Regarding the GO term associated with the cellular component for the ERT group, we found under-expressed genes that are components of keratin filaments (KRT1, KRT5, KRT6C, and KRT77), secretory granule lumen (FGA, ORM1, ORM2, SERPINF2, and TF) and components of the cell membrane microparticles circulating in the blood, blood microparticles (AMBP, FGA, GC, IGHA2, IGHG4, KRT1, ORM1, ORM2, SERPINF2, and TF) (Figure 5C).

### 3.5. Interactome Analysis

**Figure 6:**
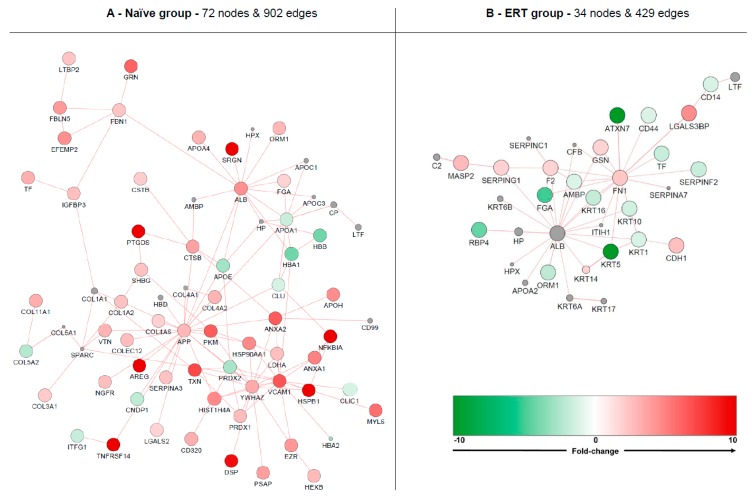
Molecular clustering based on protein-protein interactions (PPIs). Red node colour denotes an increased expression of the protein and green a decrease. Nodes displaying grey colour represent enriched molecules from GeneMania. Analysis was based on naive (**A**) and ERT groups (**B**).

Since not every protein/gene has annotated functions, and in order to cover molecules that are either poorly annotated or have no known function, protein-protein interaction clustering was performed with both datasets (naïve and ERT groups) representing their respective molecular expression using GeneMania app for Cytoscape [17,21] (Figure 6). The displayed networks from the analysis of protein-protein interactions (PPIs) allowed the identification of three highly interconnected nodes or network hubs in the naïve group: amyloid beta A4 protein (APP), 14-3-3 protein zeta/delta (YWHAZ), and serum albumin (ALB) (Figure 6A). In the ERT group ALB is one of the enriched molecules added from the PPI analysis and is shown as a hub of the network cluster. Fibronectin (FN1) is shown as a cluster hub and is overexpressed (Figure 6B).

Isolated nodes and small clusters (with less than three molecules bonding) were removed in order to highlight only relevant network features. Globally, the naïve group formed an up-regulated cluster containing 72 nodes and 902 edges. The ERT group formed a down-regulated cluster containing 34 nodes and 429 edges. The most up-regulated molecules for the naïve group cluster are serglycin (SRGN), prostaglandin-H2 D-isomerase (PTGDS), amphiregulin (AREG), tumor necrosis factor receptor superfamily member 14 (TNFRSF14), desmoplakin (DSP), heat shock protein beta-1 (HSPB1) and NF-kappa-B inhibitor alpha (NFKBIA). The most down-regulated molecules for the ERT group cluster are ataxin-7 (ATXN7) and keratin type II cytoskeletal 5 (KRT5). An enriched network with PPIs for Fabry disease containing additional data from DisGeNET can be found in Supplementary Figure S6.

### 3.6. Disease Analysis—DisGeNET

Based on the DisGeNET database score that uses supportive evidence from the literature and other disease databases to prioritise gene-disease associations, we found GLA, NAT8, NOS3, IL6, CRP, VDR, NAIP, NOS2, and ICAM1 as the top 10 more accountable associated genes related with FD (Table S5). Likewise, phenotypes and other traits more similar with FD (based on the number of shared genes) can be retrieved from DisGeNET. The top 10 disease and phenotype associations for FD are a multitude of neoplasm and carcinomas from different tissue and/or organ types, such as malignant neoplasm of breast, breast carcinoma, malignant neoplasm of prostate, liver carcinoma, Prostate carcinoma, Carcinogenesis, Hypertensive disease, Colorectal Cancer, Neoplasm metastasis, malignant neoplasm of lung (Table S6). In a similar way one can observe which genes are shared among Fabry disease with the group of kidney diseases (Table S7).

The naïve group shares more than half of the genes (from an initial gene number of 25) with malignant neoplasm of breast, breast carcinoma, non-insulin-dependent diabetes mellitus, and schizophrenia (Table 2). The ERT patients group shares more than half of the genes (from an initial gene number of 14) with asthma, obesity, Alzheimer's disease and malignant neoplasm of the breast (Table 3).

### 3.7. Regulatory Interactions

**Figure 7:**
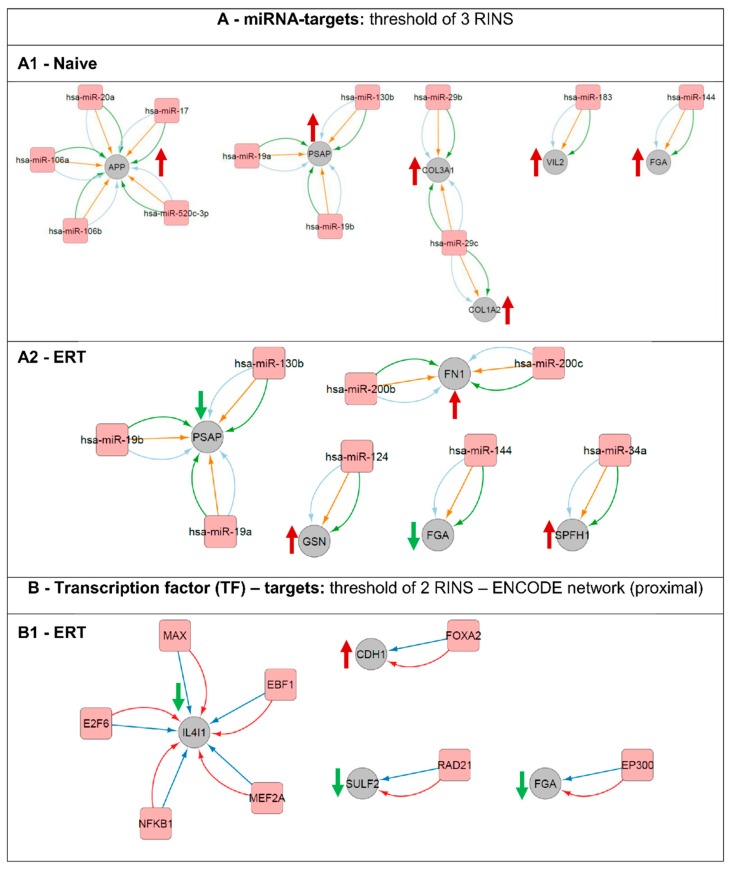
Regulatory network interactions of putative gene transcription regulators and their respective targets. Analysis was done based on miRNA-targets (**A**) and transcription factors (**B**). (**A1**) miRNA-targets for the naïve group. (**A2**) miRNA-targets for the ERT group. (**B1**) transcription factor (TF)-targets for the ERT group. An arrow’s colour and direction indicates the regulation trend of the target molecules (green: down-regulated; red: up-regulated). The naïve group regarding the TF-targets did not fulfil the minimum requirement of two RINS in the CyTargetLinker app analysis thereby is not displayed here.

Network analysis with regulatory interactions was performed using the CyTargetLinker app for Cytoscape in order to find miRNA-target, transcription factor-target or drug-target interactions for both dataset groups (naïve and ERT patients) (Figure 7). For this, regulatory data from designated Regulatory Interaction Networks (RegINs) was used. MicroRNAs (miRNAs) are a class of endogenous, short ~22 nucleotides in length, which are non-coding RNAs that play an important role as post-transcriptional regulators of gene expression. Therefore, molecules affected by the same up-stream factors are expected to display a similar expression pattern regulation, and consequently similarly modulated genes might be affected by the same up-stream events.

Here, it was possible to pinpoint potential up-stream regulators and their targets for the naïve group (Figure 7A1). Expression of amyloid beta A4 protein (APP) is being regulated by miR-20a, miR-17, miR-520c-3p, miR-106b and miR-106a. Prosaposin (PSAP) is being regulated by miR-19a, miR-130b and miR-19b. Extracellular matrix structural constituents such as the collagen alpha-1(III) chain (COL3A1) is being regulated by both miR-29b and miR-29c and the collagen alpha-2(I) chain (COL1A2) by miR-29c. Ezrin (VIL2) involved in key cytoskeletal structures of the plasma membrane is being regulated by miR-183. Fibrinogen alpha chain (FGA) is one of the primary components of blood clots and is being regulated by miR-144. Prosaposin (PSAP) and fibrinogen alpha chain (FGA) are modulators/regulators, respectively, on e.g., lipid binding and e.g., when involved in structural molecule activity. In our analysis, both proteins were found differentially expressed for the groups of naïve and ERT patients, up and down-regulated respectively. The PSAP gene expression is regulated by the following microRNAs and they are expected to be down-regulated: miR-19a, miR-19b and 130b. FGA gene expression appears to be regulated by miR-144 and by the histone acetyltransferase p300 (EP300) (Figure 7A1,A2,B1). Fibronectin (FN1) binds cell surfaces and various compounds including collagen, fibrin, heparin, DNA, and actin and is being targeted by miR-200b and miR-200c. Gelsolin (GSN), a calcium-regulated, actin-modulating protein is being up-stream regulated by miR-124. Erlin-1 (ERLIN1, SPFH1), a lipid-binding protein, is being regulated by miR-34a (Figure 7A2). Similarly, L-amino-acid oxidase (IL4I1), an enzyme that might play a role in lysosomal antigen processing and presentation, is being regulated at the transcription level by the following transcription factors: protein max (MAX), transcription factor COE1 (EBF1), myocyte-specific enhancer factor 2A (MEF2A), nuclear factor NF-kappa-B p105 subunit (NFKB1), and transcription factor E2F6 (E2F6) (Figure 7B1). Cadherin-1 (CDH1), a calcium-dependent cell adhesion protein with a strong inhibitory effect on APP, C99 and C83 production, is being regulated on the transcription level by the hepatocyte nuclear factor 3-beta (FOXA2). The extracellular sulfatase Sulf-2 (SULF2), an enzyme associated with kidney development, is being regulated by the double-strand-break repair protein rad21 homolog (RAD21) (Figure 7B1).

### 3.8. Merging Results and Overall Interpretation

**Figure 8:**
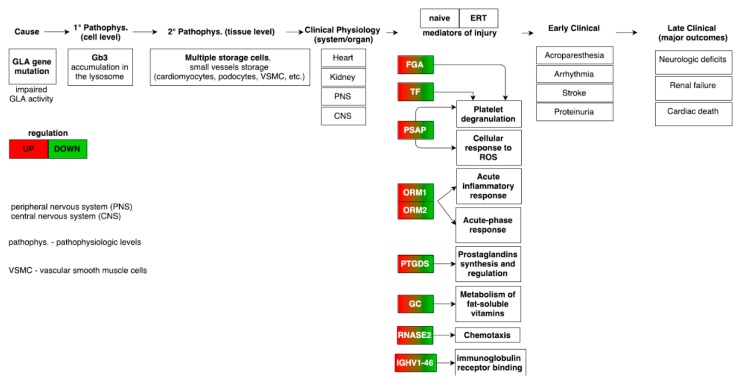
Mapping of the pathophysiologic and molecular features in Fabry-naïve and ERT groups providing the link from disease cause to final major clinical outcomes [34]. The highlighted molecular features from the meta-analysis demonstrates an opposite regulation trend in the Fabry-naïve and ERT groups.

The combination of all the results from gene ontology, pathway term clustering, interactome analysis based on PPIs, matching miRNAs to target genes, to linking genes to metabolites through the analysis of metabolic pathways, leads to the description as a whole based on the several individual datasets collected for FD. Therefore, clustering based on GO and pathway terms, combined with the mapped molecules onto pathways, has shown how the regulation of acute-phase response proteins (FGA, ORM1, ORM2, ITIH4 and SERPINA3) and the platelet degranulation (FGA, TF, ALB, PSAP, CLU, APOA1) processes were found to be up-regulated, whereas, the regulation (positive) of nitric oxide biosynthesis process (CLU and AGT) and the regulation (positive) of cholesterol esterification (APOA1 and AGT) were both found to be down-regulated (Figure 8).

## 4. Discussion

In this integrative systems biology meta-analysis, after performing gene ontology and pathway term clustering, it was found in the Fabry-naïve (non-treated) group that this involves major biological processes such as the acute inflammatory response, regulation of wound healing, extracellular matrix (ECM) remodelling, regulation of peptidase activity and cellular response to reactive oxygen species (ROS). The acute inflammatory response was found activated with all the genes belonging to cluster #1 (DEFB1, IL1RN, ITIH4, ORM1, ORM2, SERPINA3, VCAM1 and VTN) increased in expression.

Platelets are highly specialised inflammatory effector cells for hemostasis and are the first responders in case of vascular injury and endothelial disruption. They are involved in a full spectrum of processes from acute inflammation to adaptive immunity, and are actively associated with tissue remodelling [35,36,37,38].

From this analysis, we describe the process of platelet activation (C1QTNF1, COL1A2, COL3A1, FGA, GP6, HSPB1, RNASE1, YWHAZ) initiated by binding of collagen (COL1A2, and COL3A1) to the platelet glycoprotein VI (GP6) which in turn leads to signal transduction through the involvement of FcR gamma-chain, the Src kinases (e.g., Fyn/Lyn) and the linker for activation of T-cell family member 1 (LAT) adapter protein, and therefore leads to the activation of phospholipase C gamma 2 (PLCG2) [39]. Ultimately, the process leads to the activation of the ligand-binding function of integrin beta-3 to bind fibrinogen alpha chain (FGA). Subsequently platelet adhesion and aggregation is mediated, resulting in overall platelet spreading, granule secretion (degranulation), stabilisation of platelet adhesion and aggregation, and finally clot retraction.

The sub-process of granule secretion by platelets (platelet degranulation) from the GO term association analysis involves ALB, APOH, APP, FGA, ITIH4, ORM1, ORM2, PSAP, SERPINA3, SRGN, and TF and leads to exocytosis of secretory granules containing preformed mediators such as histamine and serotonin and in this way amplifies thrombus formation at sites of vascular injury by enhancing cell activation, reinforcing cell adhesion and inducing cell migration and tissue regeneration and repair. Platelet granule secretion has an important role in the pathogenesis of ischemic cardio- and cerebrovascular diseases [40,41]. Additionally, the release of prostaglandin (PTGDS catalyses the conversion of PGH2 to PGD2) mediators of the inflammation process can contribute to its resolution and promote wound healing (ANXA1, and ANXA2). Prostaglandin D2 (PGD2) is a prostaglandin involved in smooth muscle contraction/relaxation and a potent inhibitor of platelet aggregation [42].

The wound-healing process was found activated with genes belonging to t cluster #1 (ANXA1, ANXA2, APOH, C1QTNF1, FGA, and VTN) being increased in expression. Regulation of wound healing modulates the rate, frequency, or extent of the series of events that restore integrity to a damaged tissue, following an injury. Annexin A1 (ANXA1) plays an important role in the innate immune response as effector of glucocorticoid-mediated responses and regulator of the inflammatory process [43].

The process of extracellular matrix organisation (ECM) was found activated with the genes belonging to cluster #1 (ANXA2, APP, COL11A1, COL1A2, COL3A1, COL4A2, COL4A6, FBLN5, FBN1, FGA, LTBP2, MATN4, SPINT1, VCAM1, and VTN) being increased in expression. This process is of main importance for events occurring at early development and for maintenance of tissue homeostasis; its dysregulation thus leads to aberrant extracellular matrix (ECM) remodelling and ultimately to the establishment of a disease state. The disruption of the trade-off between production and degradation of ECM during a chronic tissue injury can lead to the deposition of ECM and then lead to pathological fibrosis [44]. Fibrosis can be the end-result of tissue injury, inflammation and apoptosis and is normally considered a final irreversible event. Nevertheless, in some clinical conditions, fibrosis is an early event and there is evidence that diabetic nephropathy (DN) and Fabry disease (FD) may be part of such conditions [45,46]. The vascular cell adhesion protein 1 (VCAM1) seems to have a function in leukocyte-endothelial cell adhesion. It interacts with integrins on leukocytes, and mediates adhesion and signal transduction. Their interaction with integrins could have a role in the pathophysiology of the immune response and in leukocyte chemotaxis to sites of inflammation [47].

The process of regulation of peptidase activity was found activated with the genes belonging to cluster #1 (APP, CD27, COL28A1, CSTB, ITIH4, NAIP, NGFR, PI3, SERPINA3, SPINK1, SPINT1, and VTN) being increased in expression. Enzymes with proteolytic activity can exert directly their action by degrading ECM or indirectly by activating other proteases, which in turn can then degrade ECM. This process is regulated by a complex array of activators, inhibitors and cellular receptors. Here, we describe a proteolytic-antiproteolytic imbalance due to an increase of the collagen catabolic process. This is verified by the increase in expression of collagens (COL11A1, COL1A2, COL3A1, COL4A2, and COL4A6) and cathepsins (CTSB); the latter is involved in an intricate activation cascade of proteases that leads to proteolysis, contrasting with the presence of overexpressed protease inhibitors (PI3, SERPINA3, SPINK1, SPINT1, ITIH4, and CSTB).

The process of cellular response to reactive oxygen species (ROS) was found activated with the genes belonging to cluster #1 (ANXA1, APOA4, FBLN5, PRDX1, PSAP, and TXN) being increased in expression. Free radicals react with main cellular components like proteins and lipids and in this way they are able to modify the balance of the proteolytic-antiproteolytic state,thereby damaging the cellular membrane. Globotriaosylceramide (Gb3) induces oxidative stress and increases cell adhesion molecule expression in Fabry disease (FD) endothelial cells [48].

Several studies have implicated miRNAs as potent regulators of the ECM. The miR-29 family, in our study miR-29b and miR-29c, regulates the expression of COL1A2 and COL3A genes involved in ECM remodelling. Other mechanisms that limit fibrosis are signalling mediators such as interferon-γ (IFNγ) and peroxisome proliferator activated receptor-γ (PPARγ) that exert their anti-fibrotic effects by antagonizing TGFβ signalling. Vitamin D receptor (VDR) ligands can decrease liver fibrosis by redirecting VDR to SMAD3 sites on the DNA, thereby reducing SMAD3 occupancy. This molecular competition for binding sites inhibits SMAD3-mediated transcription of ECM targets and prevents hepatic stellate cell activation in the liver [49]. The association between ECM component dysregulation and overall disease severity suggests that ECM turnover may play a role in FD pathogenesis [49,50].

Contrasting regulation of acute-phase response proteins and increased platelet degranulation of the groups of naïve (up-regulation of ORM1, ORM2, ITIH4, SERPINA3, FGA and PSAP) and ERT (down-regulation of FGA, PSAP, ORM1 and ORM2) patients (Figure 3 and Figure 6) as potential hallmarks for the distinction between naïve patients and those undergoing ERT. Moreover, proteins secreted by activated platelets can adhere to the vessel wall and promote the development of atherosclerosis and thrombosis [51]. The formation of the fibrin clot (blood coagulation cascade) pathway found in ERT patient groups can make sense, since the core source of patient injury has been attenuated with treatment by enzymatic therapy.

On the other hand, Vedder and collaborators [52] found that only minimal abnormalities in markers for platelet, endothelial activation, and coagulation activation and fibrinolysis could be established in a large cohort of Fabry disease patients. Therefore, they claimed that these abnormalities are more likely related to renal insufficiency than Fabry disease itself.

Systems biology requires comprehensive data at all molecular levels, hence using a data-driven approach based in molecular expression profiling of a disease and/or disease states could be a promising approach to finding potential body fluid disease biomarkers and consequently provide putative therapeutic targets. Nevertheless, mining the increasing data across different labs is still a great challenge which affects the efficiency of selecting useful molecular candidates, resulting in the cumulative increase of redundant and falsely identified molecules. Furthermore, these molecules will ultimately populate the increasing medical literature and will become a source for biased information and thus a burden for decision making with respect to candidate disease biomarkers.

## 5. Conclusions

From our meta-analysis we found evidence to support the hypothesis that an increased endothelial inflammatory profile exists in Fabry disease, with increased impediment of progressing through the several phases of wound healing, thus installing a prolonged inflammatory phase. In this way, increased levels of protease inhibitors, enhanced phenomena of ECM remodelling, and damage of survival and growth factors needed to achieve a complete healing are promoted. Concomitantly, reactive oxygen species (ROS) can chemically modify these vital components.

The pathogenicity of Fabry disease (FD) has been commonly attributed to the long-term and progressive deposition of globotriaosylceramide (Gb3) in the vascular endothelium; however, this initial metabolic defect triggers a cascade of molecular events that leads to endothelial dysfunction and, with the progression of the disease, may promote recurrent thrombotic events during the patient’s life.

## Figures and Tables

**Table 1:** Description of data sources used in this meta-analysis for both FD-naïve and ERT groups. EXPREF IDs contain PubMed IDs derived from the publication where data originated from. N/A: not available.

EXPREF	Total	N (case)	N (control)	Disease Case	Disease Control	Source	Detection Method	Reference
Exp25666440	23	11	12	naïve Fabry patients	ERT	urine	nanoLC-ESI-MS/MS	[25]
Exp17301227	13	N/A	N/A	6 months of ERT	before treatment (baseline)	blood	nanoLC-ESI-MS/MS	[26]
Exp18339188a	13	N/A	N/A	6 months of ERT	before treatment (baseline)	blood	microarray	[27]
Exp18339188b	13	N/A	N/A	naïve Fabry patients	healthy	blood	microarray	[27]
Exp20954982	30	20	10	Fabry	healthy	urine	MALDI-TOF MS	[28]
Exp23385635	14	8	6	Fabry	healthy	blood	MALDI-TOF MS	[29]
Exp23464927	10	N/A	N/A	12 months of ERT	before treatment (baseline)	urine	QTOF MS/MS	[30]
Exp25619383	46	32	14	Fabry	healthy	blood	LC-MS/MS	[31]
Exp21698285	124	35	89	naïve female Fabry	healthy	urine	CE-MS	[32]
Exp26490183	6	N/A	N/A	short term of ERT	before treatment (baseline)	blood	Illumina MiSeq instrument	[8]
Exp25582508	32	16	16	untreated Fabry males	healthy	urine	UPLC-ESI-TOF-MS	[33]

**Table 2:** DisGeNET disease analysis for the naïve group.

Disease Name	# Shared Genes	Gene Name
Malignant neoplasm of breast	16	AGT, ALB, APOA1, APOA4, CLU, GC, GRN, IGK, ITIH4, PSAP, PTGDS, RNASE1, SERPINA3, SLURP1, TF, YWHAZ
Breast carcinoma	15	AGT, ALB, APOA1, APOA4, CLU, GC, GRN, ITIH4, PSAP, PTGDS, RNASE1, SERPINA3, SLURP1, TF, YWHAZ
Diabetes mellitus, non-insulin-dependent	13	AGT, ALB, APOA1, APOA4, CLU, FGA, GC, GRN, HBA1, PTGDS, RNASE1, SERPINA3, TF
Schizophrenia	13	APOA1, APOH, CLU, GC, GRN, ITIH4, PSAP, PTGDS, RNASE1, SERPINA3, SHISA5, TF, YWHAZ
Hypertensive disease	12	AGT, ALB, APOA1, CLU, FGA, GC, GRN, PTGDS, RNASE1, SERPINA3, TF, YWHAZ
Liver carcinoma	12	AGT, ALB, APOA1, APOA4, APOH, CLU, FGA, GC, GRN, PTGDS, UMOD, YWHAZ
Diabetes mellitus	12	AGT, ALB, APOA1, APOA4, APOH, CLU, FGA, GC, GRN, PTGDS, UMOD, YWHAZ
Diabetes	12	AGT, ALB, APOA1, APOA4, APOH, CLU, GC, HBA1, PTGDS, SERPINA3, UMOD, YWHAZ
Atherosclerosis	12	AGT, ALB, APOA1, APOA4, APOH, CLU, GC, HBA1, PTGDS, SERPINA3, UMOD, YWHAZ
Arteriosclerosis	12	AGT, ALB, APOA1, APOH, CLU, FGA, GC, HBA1, RNASE1, SERPINA3, UMOD, YWHAZ
Alzheimer‘s disease	12	AGT, ALB, APOA1, APOA4, APOH, CLU, GC, GRN, RNASE1, SERPINA3, TF, YWHAZ
Asthma	11	AGT, APOA1, GC, IGHG1, ORM1, PSAP, PTGDS, RNASE2, SERPINA3, TF, YWHAZ
Obesity	11	AGT, ALB, APOA1, APOA4, APOH, CLU, FGA, HBA1, TF, UMOD, YWHAZ
Cardiovascular Diseases	11	AGT, ALB, APOA1, APOA4, APOH, FGA, HBA1, RNASE1, RNASE2, SERPINA3, TF
Cerebrovascular accident	11	AGT, APOA1, CLU, GC, GRN, PSAP, PTGDS, RNASE1, SERPINA3, TF, YWHAZ
Malignant neoplasm of prostate	11	AGT, ALB, APOA1, APOA4, FGA, GC, GRN, ORM1, PTGDS, TF, YWHAZ
Prostate carcinoma	11	AGT, APOA1, CLU, GC, GRN, PSAP, PTGDS, RNASE1, SERPINA3, TF, YWHAZ
Mammary Neoplasms	10	AGT, ALB, CLU, GRN, HBA1, PSAP, PTGDS, RNASE1, SLURP1, YWHAZ
Colorectal Cancer	10	AGT, ALB, APOA1, CLU, GC, ORM2, PSAP, PTGDS, RNASE1, YWHAZ
Neoplasm metastasis	10	AGT, CLU, GC, GRN, IGK, PSAP, PTGDS, RNASE1, UMOD, YWHAZ
Carcinogenesis	10	AGT, ALB, APOA1, CLU, GRN, PSAP, RNASE1, SERPINA3, TF, YWHAZ

**Table 3:** DisGeNET disease analysis for the ERT group.

Disease Name	# Shared Genes	Gene Name
Asthma	8	GC, IGHG1, ORM1, PSAP, PTGDS, RNASE2, SERPING1, TF
Obesity	7	F2, FGA, GC, PTGDS, RBP4, SERPING1, TF
Alzheimer's disease	7	AMBP, F2, GC, IGK, PSAP, PTGDS, TF
Malignant neoplasm of breast	7	F2, FGA, GC, ORM1, PTGDS, RBP4, TF
Atherosclerosis	6	AMBP, F2, FGA, GC, PTGDS, RBP4
Arteriosclerosis	6	AMBP, F2, GC, PSAP, PTGDS, TF
Breast carcinoma	6	F2, FGA, GC, PTGDS, RBP4, TF
Diabetes mellitus, non-insulin-dependent	5	AMBP, F2, FGA, GC, PTGDS, RBP4
Malignant neoplasm of prostate	5	F2, GC, ORM2, PSAP, PTGDS
Diabetes mllitus	5	AMBP, F2, FGA, GC, TF
Drug-induced liver injury	5	F2, GC, RBP4, SERPING1, TF
Cardiovascular diseases	5	GC, PSAP, PTGDS, RBP4, TF
Liver carcinoma	5	GC, PSAP, PTGDS, RBP4, TF
Colorectal cancer	4	AMBP, F2, FGA, RBP4, TF
Prostate carcinoma	4	AMBP, F2, GC, PTGDS, RBP4
melanoma	4	F2, FGA, GC, TF
Mammary neoplasms	4	F2, FGA, RNASE2, TF
Malignant neoplasm of ovary	4	F2, GC, PSAP, PTGDS
Colorectal carcinoma	4	F2, FGA, GC, RBP4
Diabetes	4	F2, GC, PTGDS, RBP4
Diabetic nephropathy	4	GC, PTGDS, RBP4, TF

## Supplementary Materials

The following are available online at www.mdpi.com/2079-9721/4/4/35/s1, Figure S1: Reactome terms representing molecular regulation for Fabry-naïve patients, Figure S2: KEGG terms representing molecular regulation for Fabry-naïve patients, Figure S3: WikiPathways terms representing molecular regulation for Fabry-naïve patients, Figure S4: Reactome terms representing molecular regulation for ERT patients, Figure S5 WikiPathways terms representing molecular regulation for ERT patients, Figure S6: Naïve group molecules plus the most relevant molecules from DisGeNET = 100 nodes and 1517 edges, Table S1: Most frequent genes/proteins found across the naïve patients datasets and their respective functional tag and regulation, Table S2: Most frequent genes/proteins found across the ERT patients datasets and their respective functional tag and regulation, Table S3: GO and pathway terms clustering from ClueGO analysis of data of the FD group, Table S4: GO and pathway terms clustering from ClueGO analysis of data of the ERT group, Table S5: Top 10 gene associations for Fabry disease, Table S6: Top 10 diseases and phenotypes that share genes with Fabry disease, Table S7: Shared genes with Fabry disease amongst the kidney diseases group.
