# Supplementary material for: Integrative Systems Biology Investigation of Fabry Disease
Source: Diseases. 2016 Nov 15;4(4):35. doi: 10.3390/diseases4040035 (PMC5456327; doi:10.3390/diseases4040035)
Supplement: Supplementary file 1 [file diseases-04-00035-s001.pdf]

Supplementary Materials: Integrative Systems Biology Investigation of Fabry Disease

Marco Fernandes and Holger Husi

1. Most Frequently Reported Genes/Proteins across the Dataspace—Naïve Patients

Table S1. Most frequent genes/proteins found across the naïve patients datasets and their respective functional tag and regulation.

Protein Name	Gene Name	PADB-KW Long	Regulation	Frequency
Ig kappa chain	IGK, SDNK1, A30	IGG: Immunoglobulin	up	4
Alpha-1-acid glycoprotein 1 precursor	ORM1, AGP1, ORM2	ENZ: enzyme, enzymatic properties	up	3
Alpha-1-acid glycoprotein 2 precursor	ORM2, AGP2, AGP-B	ENZ: enzyme, enzymatic properties	up	3
Serum albumin precursor	ALB, GIG20, GIG42	TP: transport, storage, endocytosis, exocytosis, vesicles	up	3
Apolipoprotein A-IV precursor	APOA4, ApoA-IV, APOA-IV	TP: transport, storage, endocytosis, exocytosis, vesicles	up	3
Hemoglobin subunit alpha	HBA1, HBA2, HBZ	TP: transport, storage, endocytosis, exocytosis, vesicles	down	3
Inter-alpha-trypsin inhibitor heavy chain H4 precursor	ITIH4, IHRP, ITIHL1	ENZ: enzyme, enzymatic properties	up	3
Prostaglandin-H2 D-isomerase precursor	PTGDS, PDS, PGDS2	ENZ: enzyme, enzymatic properties	up	3
Uromodulin precursor	UMOD	DIS: disease	up	3
14-3-3 protein zeta/delta	YWHAZ	SCA: scaffolder, docking, adaptor	up	2
Alpha-1-antichymotrypsin precursor	SERPINA3, AACT, GIG24	INH: inhibitor (protease, kinase, other enzymes, pathways)	up	2
Angiotensinogen	AGT, SERPINA8	SIG: signalling	down	2
Apolipoprotein A-I precursor	APOA1, A175P	TP: transport, storage, endocytosis, exocytosis, vesicles	down	2
Beta-2-glycoprotein 1 precursor	APOH, B2G1	UK: unknown	up	2
Clusterin precursor	CLU, APOJ, CLI	CHA: chaperone, chaperonin	down	2
Fibrinogen alpha/alpha-E chain precursor	FGA	MOD: modulator, regulator	up	2
Granulins precursor	GRN	SIG: signalling	up	2
Ribonuclease pancreatic precursor	RNASE1, RIB1, RNS1	ENZ: enzyme, enzymatic properties	up	2
Nonsecretory ribonuclease precursor	RNASE2, EDN, RNS2	ENZ: enzyme, enzymatic properties	up	2
Proactivator polypeptide precursor	PSAP, GLBA, SAP1	MOD: modulator, regulator	up	2
Protein SHISA-5	SHISA5, SCOTIN	SIG: signalling	up	2
Secreted Ly-6/uPAR related protein 1 precursor	SLURP1, ARS	SIG: signalling	up	2
Vitamin D-binding protein precursor	GC	CS: Cell shape (cytoskeleton, cell adhesion, morphology, cell junction, cellular structures, extracellular matrix)	up	2
Immunoglobulin G1 Fab heavy chain variable region	IGH, VH1, IGHG1	IGG: Immunoglobulin	up	2
Serotransferrin precursor	TF, PRBPO1400	TP: transport, storage, endocytosis, exocytosis, vesicles	up	2

2. Most Frequently Reported Genes/Proteins across the Dataspace—ERT Patients

Table S2. Most frequent genes/proteins found across the ERT patients datasets and their respective functional tag and regulation.

Protein Name	Gene Name	PADB-KW Long	Regulation	Frequency
Ig kappa chain	IGK, SDNK1, A30	IGG: Immunoglobulin	up	4
Alpha-1-acid glycoprotein 1 precursor	ORM1, AGP1, ORM2	ENZ: enzyme, enzymatic properties	down	3
Alpha-1-acid glycoprotein 2 precursor	ORM2, AGP2, AGP-B	ENZ: enzyme, enzymatic properties	down	3
Prostaglandin-H2 D-isomerase precursor	PTGDS, PDS, PGDS2	ENZ: enzyme, enzymatic properties	down	3
AMBP protein precursor	AMBP, ITIL, HCP	INH: inhibitor (protease, kinase, other enzymes, pathways)	down	2
Fibrinogen alpha/alpha-E chain precursor	FGA	MOD: modulator, regulator	down	2
Serine/cysteine proteinase inhibitor clade G member 1	SERPING1, C1IN, C1NH	INH: inhibitor (protease, kinase, other enzymes, pathways)	up	2
Retinol binding protein 4, plasma	RBP4, PRO2222, PRBP	TP: transport, storage, endocytosis, exocytosis, vesicles	down	2
Nonsecretory ribonuclease precursor	RNASE2, EDN, RNS2	ENZ: enzyme, enzymatic properties	down	2
Proactivator polypeptide precursor	PSAP, GLBA, SAP1	MOD: modulator, regulator	down	2
Prothrombin precursor	F2	ENZ: enzyme, enzymatic properties	up	2
Vitamin D-binding protein precursor	GC	CS: Cell shape (cytoskeleton, cell adhesion, morphology, cell junction, cellular structures, extracellular matrix)	down	2
Immunoglobulin G1 Fab heavy chain variable region	IGH, VH1, IGHG1	IGG: Immunoglobulin	down	2
Serotransferrin precursor	TF, PRO1400	TP: transport, storage, endocytosis, exocytosis, vesicles	down	2

3. Analysis with All Reported Features—Term Clustering Naïve Patients—Gene Ontology (GO) Plus Associated Genes with Processes and Pathways

Reactome

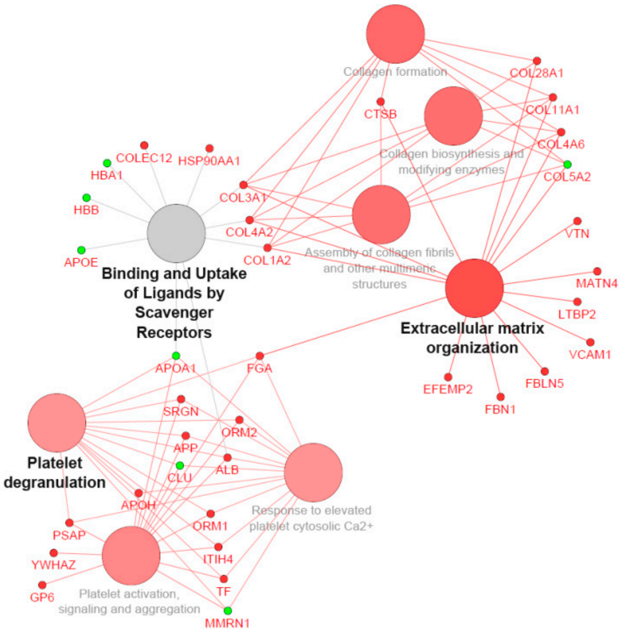

Figure S1. Reactome terms representing molecular regulation for Fabry naïve patients.

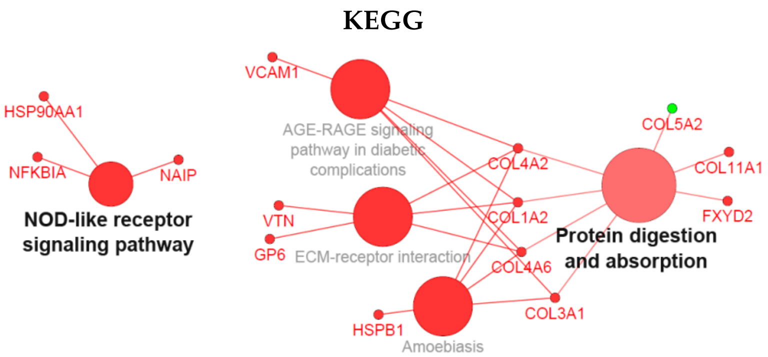

Figure S2. KEGG terms representing molecular regulation for Fabry naïve patients.

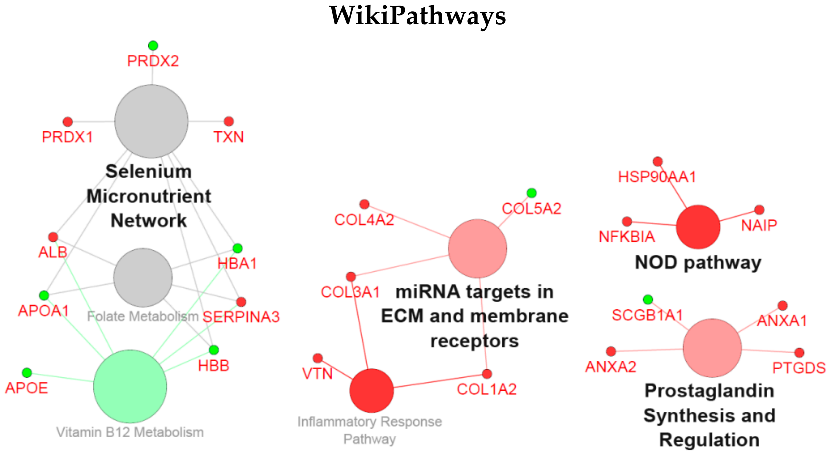

Figure S3. WikiPathways terms representing molecular regulation for Fabry naïve patients.

4. Analysis with All Reported Features—Term Clustering ERT Patients—Gene Ontology (GO) Plus Associated Genes with Processes and Pathways

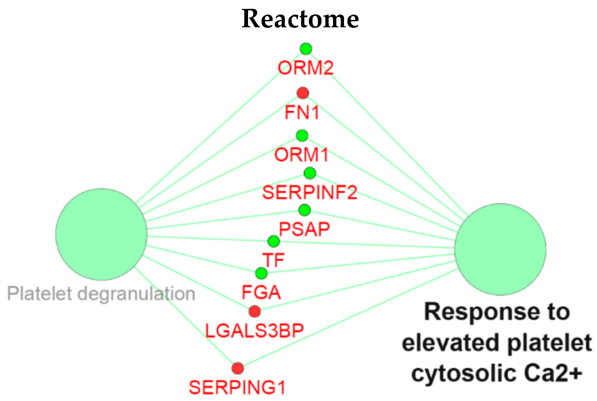

Figure S4. Reactome terms representing molecular regulation for ERT patients.

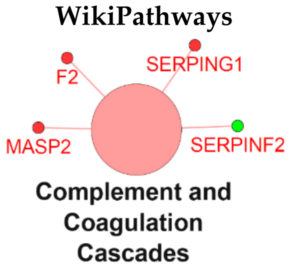

Figure S5. WikiPathways terms representing molecular regulation for ERT patients.

Table S3. GO and pathway terms clustering from ClueGO analysis of data of the FD group. Cluster #1 and cluster #2 correspond respectively to up-regulated and down-regulated molecules.

GO Term	Term BF-*p*-Value	Group BF-*p*-Value	GO Groups	% Associated Genes	Cluster	Genes Cluster #1	Genes Cluster #2	% Genes Cluster #1	% Genes Cluster #2
**BP: Biological Process**									
acute inflammatory response	17.0E-6	17.0E-6	Group 0	5.06	Specific for Cluster #1	[DEFB1, IL1RN, ITIH4, ORM1, ORM2, SERPINA3, VCAM1, VTN]		100.00	0.00
platelet degranulation	300.0E-15	160.0E-15	Group 1	12.17	Specific for Cluster #1	[ALB, APOH, APP, FGA, ITIH4, ORM1, ORM2, PSAP, SERPINA3, SRGN, TF]	[APOA1, CLU, MMRN1]	78.57	21.43
multicellular organism metabolic process	1.4E-6	88.0E-9	Group 2	6.77	Specific for Cluster #1	[APOA4, COL11A1, COL1A2, COL3A1, COL4A2, COL4A6, CTSB]	[COL5A2, PRTN3]	77.78	22.22
multicellular organism catabolic process	17.0E-9	88.0E-9	Group 2	11.84	Specific for Cluster #1	[APOA4, COL11A1, COL1A2, COL3A1, COL4A2, COL4A6, CTSB]	[COL5A2, PRTN3]	77.78	22.22
multicellular organismal macromolecule metabolic process	4.8E-6	88.0E-9	Group 2	6.78	Specific for Cluster #1	[COL11A1, COL1A2, COL3A1, COL4A2, COL4A6, CTSB]	[COL5A2, PRTN3]	75.00	25.00
response to amino acid	14.0E-6	88.0E-9	Group 2	6.73	Specific for Cluster #1	[C1QTNF1, COL1A2, COL3A1, COL4A6, MUC1, NAIP]	[COL5A2]	85.71	14.29
collagen catabolic process	120.0E-9	88.0E-9	Group 2	11.43	Specific for Cluster #1	[COL11A1, COL1A2, COL3A1, COL4A2, COL4A6, CTSB]	[COL5A2, PRTN3]	75.00	25.00
collagen metabolic process	4.1E-6	88.0E-9	Group 2	7.08	Specific for Cluster #1	[COL11A1, COL1A2, COL3A1, COL4A2, COL4A6, CTSB]	[COL5A2, PRTN3]	75.00	25.00
regulation of wound healing	13.0E-6	10.0E-6	Group 3	5.76	Specific for Cluster #1	[ANXA1, ANXA2, APOH, C1QTNF1, FGA, VTN]	[APOE, MYLK]	75.00	25.00
negative regulation of peptidase activity	17.0E-9	9.4E-9	Group 4	5.32	Specific for Cluster #1	[APP, CD27, COL28A1, CSTB, ITIH4, NAIP, NGFR, PI3, SERPINA3, SPINK1, SPINT1, VTN]	[AGT, GPC3]	85.71	14.29
negative regulation of endopeptidase activity	91.0E-9	9.4E-9	Group 4	5.16	Specific for Cluster #1	[APP, CD27, COL28A1, CSTB, ITIH4, NAIP, NGFR, PI3, SERPINA3, SPINK1, SPINT1, VTN]	[AGT]	92.31	7.69
platelet activation	140.0E-9	79.0E-9	Group 5	6.36	Specific for Cluster #1	[C1QTNF1, COL1A2, COL3A1, FGA, GP6, HSPB1, RNASE1, YWHAZ]	[APOE, CLIC1, HBB]	72.73	27.27
cellular response to reactive oxygen species	39.0E-6	39.0E-6	Group 6	4.90	Specific for Cluster #1	[ANXA1, APOA4, FBLN5, PRDX1, PSAP, TXN]	[PRDX2]	85.71	14.29

Table S3. *Cont.*

GO Term	Term BF-*p*-Value	Group BF-*p*-Value	GO Groups	% Associated Genes	Cluster	Genes Cluster #1	Genes Cluster #2	% Genes Cluster #1	% Genes Cluster #2
extracellular structure organization	1.3E-9	700.0E-12	Group 7	4.86	Specific for Cluster #1	[ANXA2, APP, COL11A1, COL1A2, COL3A1, COL4A2, COL4A6, FBLN5, FBN1, FGA, LTBP2, MATN4, SPINT1, VCAM1, VTN]	[AGT, COL5A2]	88.24	11.76
extracellular matrix organization	1.3E-9	700.0E-12	Group 7	4.87	Specific for Cluster #1	[ANXA2, APP, COL11A1, COL1A2, COL3A1, COL4A2, COL4A6, FBLN5, FBN1, FGA, LTBP2, MATN4, SPINT1, VCAM1, VTN]	[AGT, COL5A2]	88.24	11.76
**MF: Molecular Function**									
serine-type endopeptidase inhibitor activity	300.0E-9	300.0E-9	Group 0	8.08	Specific for Cluster #1	[APP, COL28A1, ITIH4, PI3, SERPINA3, SPINK1, SPINT1]	[AGT]	87.50	12.50
peptidase inhibitor activity	9.2E-9	9.2E-9	Group 1	6.35	Specific for Cluster #1	[APP, CD27, COL28A1, CSTB, ITIH4, NAIP, PI3, SERPINA3, SPINK1, SPINT1]	[AGT, GPC3]	83.33	16.67
CC									
extracellular matrix	2.6E-21	1.4E-21	Group 0	5.90	Specific for Cluster #1	[ANXA2, APOH, COL11A1, COL1A2, COL28A1, COL3A1, COL4A2, COL4A6, DSP, EFEMP2, FBLN5, FBN1, HSP90AA1, HSPB1, LGALS1, LTBP2, MYL6, PI3, PKM, PRDX1, RNASE1, TGM4, UMOD, VTN]	[APOE, CLU, COL5A2, GPC3, MMRN1, PCSK6, PRTN3, SPARCL1]	75.00	25.00
melanosome	84.0E-6	84.0E-6	Group 1	5.36	Specific for Cluster #1	[ANXA2, CTSB, HSP90AA1, PRDX1, RNASE1, YWHAZ]		100.00	0.00
vacuolar lumen	470.0E-9	230.0E-9	Group 2	7.14	Specific for Cluster #1	[APP, CTSB, GC, GLB1, HEXB, HSP90AA1, PSAP]	[B2M, GPC3]	77.78	22.22
lysosomal lumen	8.9E-6	230.0E-9	Group 2	7.22	Specific for Cluster #1	[CTSB, GC, GLB1, HEXB, HSP90AA1, PSAP]	[GPC3]	85.71	14.29
extracellular matrix component	270.0E-12	130.0E-12	Group 3	9.16	Specific for Cluster #1	[ANXA2, COL11A1, COL1A2, COL28A1, COL3A1, COL4A2, COL4A6, EFEMP2, FBLN5, FBN1, VTN]	[COL5A2]	91.67	8.33
basement membrane	6.8E-6	130.0E-12	Group 3	7.07	Specific for Cluster #1	[ANXA2, COL28A1, COL4A2, COL4A6, EFEMP2, FBN1, VTN]		100.00	0.00

Table S3. *Cont.*

GO Term	Term BF-*p*-Value	Group BF-*p*-Value	GO Groups	% Associated Genes	Cluster	Genes Cluster #1	Genes Cluster #2	% Genes Cluster #1	% Genes Cluster #2
cytoplasmic membrane-bounded vesicle lumen	230.0E-21	110.0E-21	Group 4	15.52	Specific for Cluster #1	[ALB, APOH, APP, C1QTNF1, FGA, HSP90AA1, ITIH4, ORM1, ORM2, SERPINA3, SRGN, TF]	[APOA1, APOE, CLU, HBA1, HBB, MMRN1]	66.67	33.33
secretory granule lumen	8.4E-15	110.0E-21	Group 4	14.74	Specific for Cluster #1	[ALB, APOH, APP, C1QTNF1, FGA, ITIH4, ORM1, ORM2, SERPINA3, SRGN, TF]	[APOA1, CLU, MMRN1]	78.57	21.43
platelet alpha granule lumen	650.0E-12	110.0E-21	Group 4	15.25	Specific for Cluster #1	[ALB, APP, FGA, ORM1, ORM2, SERPINA3, SRGN]	[CLU, MMRN1]	77.78	22.22
**Reactome**									
Extracellular matrix organization	15.0E-9	6.0E-9	Group 0	5.56	Specific for Cluster #1	[COL11A1, COL1A2, COL28A1, COL3A1, COL4A2, COL4A6, CTSB, EFEMP2, FBLN5, FBN1, FGA, LTBP2, MATN4, VCAM1, VTN]	[COL5A2]	93.75	6.25
Collagen formation	2.0E-6	6.0E-9	Group 0	8.99	Specific for Cluster #1	[COL11A1, COL1A2, COL28A1, COL3A1, COL4A2, COL4A6, CTSB]	[COL5A2]	87.50	12.50
Collagen biosynthesis and modifying enzymes	1.7E-6	6.0E-9	Group 0	10.45	Specific for Cluster #1	[COL11A1, COL1A2, COL28A1, COL3A1, COL4A2, COL4A6]	[COL5A2]	85.71	14.29
Assembly of collagen fibrils and other multimeric structures	1.2E-6	6.0E-9	Group 0	12.96	Specific for Cluster #1	[COL11A1, COL1A2, COL3A1, COL4A2, COL4A6, CTSB]	[COL5A2]	85.71	14.29
Binding and Uptake of Ligands by Scavenger Receptors	11.0E-12	4.2E-12	Group 1	24.39	Specific for Cluster #1	[ALB, COL1A2, COL3A1, COL4A2, COLEC12, HSP90AA1]	[APOA1, APOE, HBA1, HBB]	60.00	40.00
Platelet degranulation	380.0E-12	12.0E-9	Group 2	10.32	Specific for Cluster #1	[ALB, APOH, APP, FGA, ITIH4, ORM1, ORM2, PSAP, SRGN, TF]	[APOA1, CLU, MMRN1]	76.92	23.08
Platelet activation, signaling and aggregation	49.0E-9	12.0E-9	Group 2	5.47	Specific for Cluster #1	[ALB, APOH, APP, FGA, GP6, ITIH4, ORM1, ORM2, PSAP, SRGN, TF, YWHAZ]	[APOA1, CLU, MMRN1]	80.00	20.00
Response to elevated platelet cytosolic Ca2+	540.0E-12	12.0E-9	Group 2	9.92	Specific for Cluster #1	[ALB, APOH, APP, FGA, ITIH4, ORM1, ORM2, PSAP, SRGN, TF]	[APOA1, CLU, MMRN1]	76.92	23.08
**KEGG**									
NOD-like receptor signaling pathway	10.0E-3	10.0E-3	Group 0	5.26	Specific for Cluster #1	[HSP90AA1, NAIP, NFKBIA]		100.00	0.00
African trypanosomiasis	820.0E-6	330.0E-6	Group 1	11.43	Specific for Cluster #2	[VCAM1]	[APOA1, HBA1, HBB]	25.00	75.00
ECM-receptor interaction	1.9E-3	64.0E-6	Group 2	6.10	Specific for Cluster #1	[COL1A2, COL4A2, COL4A6, GP6, VTN]		100.00	0.00

Table S3. *Cont.*

GO Term	Term BF-*p*-Value	Group BF-*p*-Value	GO Groups	% Associated Genes	Cluster	Genes Cluster #1	Genes Cluster #2	% Genes Cluster #1	% Genes Cluster #2
AGE-RAGE signaling pathway in diabetic complications	2.5E-3	64.0E-6	Group 2	4.95	Specific for Cluster #1	[COL1A2, COL3A1, COL4A2, COL4A6, VCAM1]		100.00	0.00
Protein digestion and absorption	40.0E-6	64.0E-6	Group 2	7.78	Specific for Cluster #1	[COL11A1, COL1A2, COL3A1, COL4A2, COL4A6, FXYD2]	[COL5A2]	85.71	14.29
Amoebiasis	3.5E-3	64.0E-6	Group 2	5.00	Specific for Cluster #1	[COL1A2, COL3A1, COL4A2, COL4A6, HSPB1]		100.00	0.00
**WikiPathwayss**									
NOD pathway	8.1E-3	8.1E-3	Group 0	7.32	Specific for Cluster #1	[HSP90AA1, NAIP, NFKBIA]		100.00	0.00
Selenium Micronutrient Network	17.0E-6	9.7E-6	Group 1	8.99	None Specific Cluster	[ALB, PRDX1, SERPINA3, TXN]	[APOA1, HBA1, HBB, PRDX2]	50.00	50.00
Vitamin B12 Metabolism	72.0E-6	9.7E-6	Group 1	11.54	Specific for Cluster #2	[ALB, SERPINA3]	[APOA1, APOE, HBA1, HBB]	33.33	66.67
Folate Metabolism	2.1E-3	9.7E-6	Group 1	7.46	Specific for Cluster #2	[ALB, SERPINA3]	[APOA1, HBA1, HBB]	40.00	60.00
miRNA targets in ECM and membrane receptors	2.2E-3	950.0E-6	Group 2	9.76	Specific for Cluster #1	[COL1A2, COL3A1, COL4A2]	[COL5A2]	75.00	25.00
Inflammatory Response Pathway	6.7E-3	950.0E-6	Group 2	10.00	Specific for Cluster #1	[COL1A2, COL3A1, VTN]		100.00	0.00
Prostaglandin Synthesis and Regulation	1.0E-3	650.0E-6	Group 3	13.33	Specific for Cluster #1	[ANXA1, ANXA2, PTGDS]	[SCGB1A1]	75.00	25.00

Table S4. GO and pathway terms clustering from ClueGO analysis of data of the ERT group. Cluster #1 and cluster #2 correspond respectively to up-regulated and down-regulated molecules.

GO Term	Term BF-*p* Value	Group BF-*p* Value	GO Groups	% Associated Genes	Cluster	Genes Cluster #1	Genes Cluster #2	%Genes Cluster #1	%Genes Cluster #2
**BP: Biological Process**									
platelet degranulation	94.0E-12	47.0E-12	Group 0	7.83	Specific for Cluster #2	[FN1, LGALS3BP, SERPING1]	[FGA, ORM1, ORM2, PSAP, SERPINF2, TF]	33.33	66.67
acute-phase response	930.0E-9	930.0E-9	Group 1	8.47	Specific for Cluster #2	[F2, FN1]	[ORM1, ORM2, SERPINF2]	40.00	60.00
negative regulation of coagulation	1.1E-6	1.1E-6	Group 2	8.77	Specific for Cluster #2	[F2, SERPING1]	[FGA, KRT1, SERPINF2]	40.00	60.00
negative regulation of hemostasis	980.0E-9	1.1E-6	Group 2	9.62	Specific for Cluster #2	[F2, SERPING1]	[FGA, KRT1, SERPINF2]	40.00	60.00
negative regulation of blood coagulation	980.0E-9	1.1E-6	Group 2	9.62	Specific for Cluster #2	[F2, SERPING1]	[FGA, KRT1, SERPINF2]	40.00	60.00
negative regulation of wound healing	820.0E-9	1.1E-6	Group 2	7.58	Specific for Cluster #2	[F2, SERPING1]	[FGA, KRT1, SERPINF2]	40.00	60.00
fibrinolysis	83.0E-9	1.1E-6	Group 2	16.13	Specific for Cluster #2	[F2, SERPING1]	[FGA, KRT1, SERPINF2]	40.00	60.00
**MF: Molecular Function**									
interleukin-1 receptor activity	660.0E-6	530.0E-6	Group 0	28.57	Specific for Cluster #1	[IL18R1, IL1R1]		100.00	0.00
scavenger receptor activity	6.4E-3	6.4E-3	Group 1	4.26	Specific for Cluster #1	[LGALS3BP, TINAGL1]		100.00	0.00
isoprenoid binding	11.0E-3	11.0E-3	Group 2	4.55	Specific for Cluster #2	[]	[PTGDS, RBP4]	0.00	100.00
retinoid binding	16.0E-3	11.0E-3	Group 2	4.65	Specific for Cluster #2	[]	[PTGDS, RBP4]	0.00	100.00
immunoglobulin receptor binding	9.2E-3	6.9E-3	Group 3	7.14	Specific for Cluster #2	[]	[IGHA2, IGHG4]	0.00	100.00
**CC: Cellular Component**									
keratin filament	4.1E-6	4.1E-6	Group 0	5.10	Specific for Cluster #2	[KRT14]	[KRT1, KRT5, KRT6C, KRT77]	20.00	80.00
vesicle lumen	1.6E-9	2.8E-9	Group 1	6.84	Specific for Cluster #2	[FN1, LGALS3BP, SERPING1]	[FGA, ORM1, ORM2, SERPINF2, TF]	37.50	62.50
cytoplasmic membrane-bounded vesicle lumen	1.8E-9	2.8E-9	Group 1	6.90	Specific for Cluster #2	[FN1, LGALS3BP, SERPING1]	[FGA, ORM1, ORM2, SERPINF2, TF]	37.50	62.50
secretory granule lumen	440.0E-12	2.8E-9	Group 1	8.42	Specific for Cluster #2	[FN1, LGALS3BP, SERPING1]	[FGA, ORM1, ORM2, SERPINF2, TF]	37.50	62.50

Table S4. *Cont.*

GO Term	Term BF-*p* Value	Group BF-*p* Value	GO Groups	% Associated Genes	Cluster	Genes Cluster #1	Genes Cluster #2	%Genes Cluster #1	%Genes Cluster #2
platelet alpha granule	83.0E-9	2.8E-9	Group 1	7.50	Specific for Cluster #2	[FN1, SERPING1]	[FGA, ORM1, ORM2, SERPINF2]	33.33	66.67
platelet alpha granule lumen	19.0E-9	2.8E-9	Group 1	10.17	Specific for Cluster #2	[FN1, SERPING1]	[FGA, ORM1, ORM2, SERPINF2]	33.33	66.67
blood microparticle	56.0E-21	24.0E-21	Group 2	9.93	Specific for Cluster #2	[F2, FN1, GSN, LGALS3BP, SERPING1]	[AMBP, FGA, GC, IGHA2, IGHG4, KRT1, ORM1, ORM2, SERPINF2, TF]	33.33	66.67
**Reactome**									
Platelet degranulation	86.0E-12	61.0E-12	Group 0	7.14	Specific for Cluster #2	[FN1, LGALS3BP, SERPING1]	[FGA, ORM1, ORM2, PSAP, SERPINF2, TF]	33.33	66.67
Response to elevated platelet cytosolic Ca^{2+}	61.0E-12	61.0E-12	Group 0	6.87	Specific for Cluster #2	[FN1, LGALS3BP, SERPING1]	[FGA, ORM1, ORM2, PSAP, SERPINF2, TF]	33.33	66.67
**WikiPathways**									
Complement and Coagulation Cascades	60.0E-6	60.0E-6	Group 0	6.78	Specific for Cluster #1	[F2, MASP2, SERPING1]	[SERPINF2]	75.00	25.00

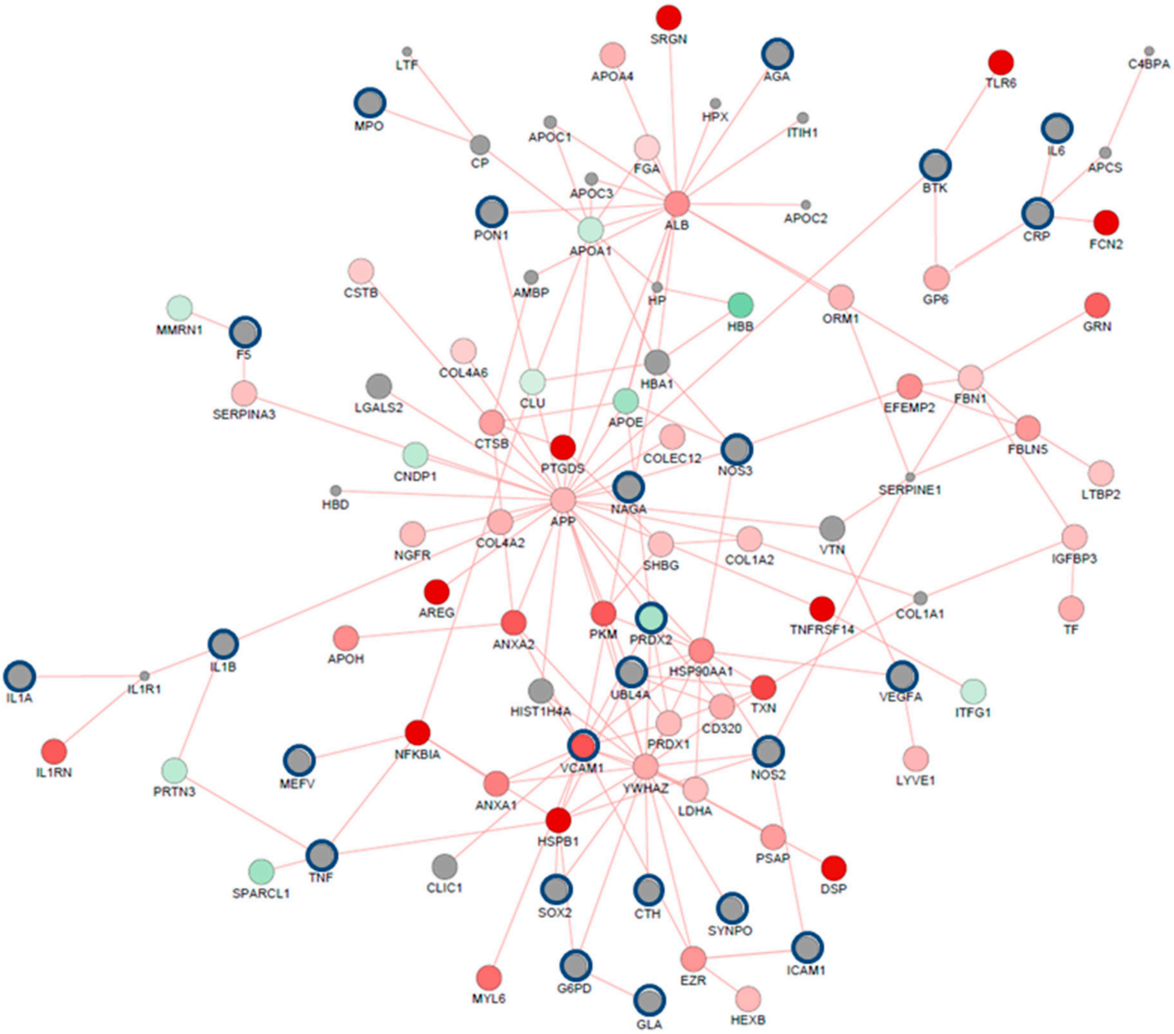

Figure S6. Naïve group molecules plus the most relevant molecules from DisGeNET—100 nodes & 1517 edges.

5. Disease Analysis—DisGeNET

Table S5. Top 10 gene associations for Fabry disease.

Symbol	Gene Name	Pathway	Panther Classification	Score	#PMIDs	#SNPs	#Diseases
GLA	galactosidase, alpha	Metabolism	null	0.665	258	23	41
NAT8	*N*-acetyltransferase 8 (GCN5-related, putative)	Metabolism of proteins	null	0.008	30	0	57
NOS3	nitric oxide synthase 3 (endothelial cell)	Hemostasis; Immune System; Metabolism; Signal Transduction	null	0.008	4	0	485
IL6	interleukin 6	Cellular responses to stress; Immune System; Signal Transduction	signalling molecule	0.005	2	0	1260
CRP	C-reactive protein, pentraxin-related	Immune System	defense/immunity protein	0.005	2	0	437

Table S5. *Cont.*

Symbol	Gene Name	Pathway	Panther Classification	Score	#PMIDs	#SNPs	#Diseases
VDR	vitamin D (1,25-dihydroxyvitamin D3) receptor	Gene Expression	transcription factor; receptor; nucleic acid binding	0.005	2	0	352
NAIP	NLR family, apoptosis inhibitory protein	null	enzyme modulator	0.003	1	0	24
NOS2	nitric oxide synthase 2, inducible	Hemostasis; Immune System	null	0.003	1	0	438
ICAM1	intercellular adhesion molecule 1	Extracellular matrix organization; Immune System	cell adhesion molecule; signalling molecule	0.003	1	0	456
SELE	selectin E	Hemostasis	cell adhesion molecule; transfer/carrier protein; protease; hydrolase; defense/immunity protein; receptor	0.003	1	0	222

Table S6. Top 10 diseases and phenotypes that share genes with Fabry disease.

Disease Name	Type	MeSH Disease Class	#Shared Genes
Malignant neoplasm of breast	disease	Neoplasms; Skin and Connective Tissue Diseases	35
Breast Carcinoma	disease	Neoplasms	34
Malignant neoplasm of prostate	disease	Neoplasms; Male Urogenital Diseases	33
Liver carcinoma	disease	Neoplasms; Digestive System Diseases	32
Prostate carcinoma	disease	Neoplasms	31
Carcinogenesis	phenotype	Neoplasms; Pathological Conditions, Signs and Symptoms	29
Hypertensive disease	disease	Cardiovascular Diseases	29
Colorectal Cancer	disease	Neoplasms; Digestive System Diseases	29
Neoplasm Metastasis	phenotype	Neoplasms; Pathological Conditions, Signs and Symptoms	29
Malignant neoplasm of lung	disease	Neoplasms; Respiratory Tract Diseases	29

Table S7. Shared genes with Fabry disease amongst the Kidney Diseases group (25 shared genes).

Symbol	Gene Name	Panther Classification	Pathway/Process
IL1A	interleukin 1, alpha	signaling molecule	Cellular responses to stress; Immune System
VCAM1	vascular cell adhesion molecule 1	cell adhesion molecule; phosphatase; hydrolase; defense/immunity protein; receptor	Extracellular matrix organization; Immune System
PON1	paraoxonase 1		
IL1B	interleukin 1, beta	signaling molecule	Immune System
TNF	tumor necrosis factor	signaling molecule	Developmental Biology; Immune System; Signal Transduction
AGT	angiotensinogen (serpin peptidase inhibitor, clade A, member 8)	enzyme modulator	Metabolism; Metabolism of proteins; Signal Transduction
NOS3	nitric oxide synthase 3 (endothelial cell)		Hemostasis; Immune System; Metabolism; Signal Transduction
NAT8	*N*-acetyltransferase 8 (GCN5-related, putative)		Metabolism of proteins
MGP	matrix Gla protein	signaling molecule; calcium-binding protein; structural protein; extracellular matrix protein	

Table S7. *Cont.*

Symbol	Gene Name	Panther Classification	Pathway/Process
G6PD	glucose-6-phosphate dehydrogenase	oxidoreductase	Gene Expression; Metabolism
ACE	angiotensin I converting enzyme		Metabolism of proteins
NOS2	nitric oxide synthase 2, inducible		Hemostasis; Immune System
MPO	myeloperoxidase	oxidoreductase	
VEGFA	vascular endothelial growth factor A	signaling molecule	Cellular responses to stress; Developmental Biology; Hemostasis; Signal Transduction
IL10	interleukin 10	signaling molecule	
F5	coagulation factor V (proaccelerin, labile factor)	cell adhesion molecule; transfer/carrier protein; transporter; protease; enzyme modulator; oxidoreductase; hydrolase; signali…	Hemostasis; Metabolism of proteins; Vesicle-mediated transport
ICAM1	intercellular adhesion molecule 1	cell adhesion molecule; signaling molecule	Extracellular matrix organization; Immune System
GCNT2	glucosaminyl (*N*-acetyl) transferase 2, I-branching enzyme (I blood group)	transferase	
UMOD	uromodulin		
VDR	vitamin D (1,25-dihydroxyvitamin D3) receptor	transcription factor; receptor; nucleic acid binding	Gene Expression
IL6	interleukin 6	signaling molecule	Cellular responses to stress; Immune System; Signal Transduction
KCNN4	potassium channel, calcium activated intermediate/small conductance subfamily N alpha, member 4		Neuronal System
TBC1D9	TBC1 domain family, member 9 (with GRAM domain)	enzyme modulator; hydrolase	
ABCB1	ATP-binding cassette, sub-family B (MDR/TAP), member 1	transporter	Metabolism; Transmembrane transport of small molecules
CRP	C-reactive protein, pentraxin-related	defense/immunity protein	Immune System
